# Association of the Myocilin Gene Polymorphism With Primary Open Angle Glaucoma

**Published:** 2019

**Authors:** Akbar DERAKHSHAN, Jalil TAVAKKOL-AFSHARI, Javad SADEGHI Allah Abadi, Mohammad-Reza ANSARI-ASTANEH, Ali Reza DERAKHSHAN, Amin Reza NIKPOOR, Saeed SHOKOOHI RAD

**Affiliations:** 1 Eye Research Center, Mashhad University of Medical Sciences, Mashhad, Iran; 2 Immunogenetic and Cell Culture Department, Immunology Research Center, School of Medicine, Mashhad University of Medical Sciences, Mashhad, Iran; 3 Department of Allergy and Immunology, School of Medicine, Mashhad University of Medical Sciences, Mashhad, Iran; 4 School of Traditional Medicine, Student Research Committee, Shahid Beheshti University of Medical Sciences, Tehran, Iran; 5 Department of Immunology, Faculty of Medicine, Hormozgan University of Medical Sciences, Bandar Abbas, Iran.

**Keywords:** PCR, Myocilin gene, Mutation, Primary Open Angle Glaucoma

## Abstract

Glaucoma is the second cause of irreversible blindness, and the Primary Open Angle Glaucoma (POAG) subtype is the most common type of glaucoma. It has been shown that genetic mutations increase the risk of POAG used for early detection. The aim of the current study was to determine the association between genetic variations of Myocilin (MYOC) gene and susceptibility to POAG in the Iranian population. This case-control study was conducted on patients with POAG, referred to Khatam-al Anbia Eye Hospital, Mashhad, Iran. The control group was selected from healthy patients with a refractive disorder, who had referred to this hospital. After extracting the DNA from the whole blood sample, the Polymerase Chain Reaction-Single-Strand Conformation Polymorphisms (PCR-SSCP) method was used to discriminate variability in sequences in three exons of MYOC gene locus, known as GLC1A. Clinical characteristics of the subjects, comprised of visual acuity, Cup to Disc Ratio (CDR), and Intra-Ocular Pressure (IOP) were statistically compared between the wild and mutant type of the MYOC gene using independent samples t-test, Chi-square, and logistic regression test with SPSS version 15.0 software. P-values of < 0.05 were considered significant. One hundred and forty participants (75.1% males) were studied in two groups of case (n = 70) and control (n = 70). The frequency of mutant alleles in patients and healthy groups was statistically significant (40% versus 11.5%, Odd’s Ratio (OR): 5.1, CI 95% for OR: 2.1 to 12.4, P-value < 0.001). Also, the detected mutation in the case group was significantly higher in exon 1 and 3 (15.7% versus 0%, P-value = 0.001, and 11.5% versus 2.8%, P-value = 0.049, respectively). Based on the result of the current study, it seems that the MYOC gene polymorphisms increased the risk of POAG in the Iranian population.

## INTRODUCTION

Glaucoma refers to a group of diseases associated with optic neuropathy and decreased vision. Glaucoma affects more than 65 million people, worldwide, and is the second most common cause of blindness [1]. By affecting 70% of glaucomatous patients among Caucasians, Primary Open Angle Glaucoma (POAG) has been considered as the most common type of glaucoma at age 40 years and older [2]. Genetic analysis has identified more than ten loci linked to POAG, attesting to its high genetic heterogeneity [3-6]. Genes at three of these loci have been identified, including Myocilin, Optineurin, and WD Repeat-containing protein 36 (WDR36). Mutations in Myocilin (MYOC) have been observed more frequently than mutations in OPTN and WDR36, although most cases of glaucoma do not involve these genetic mutations. Furthermore, MYOC or Trabecular Meshwork Inducible Glucocorticoid Response (TIGR) is a protein, which is encoded by the MYOC gene in humans. Mutations in MYOC are a major cause of glaucoma and have been discovered in 3% to 4% of patients with POAG (7). The MYOC gene locus (known as Glaucoma 1A or GLC1A), was mapped to chromosome 1q23. Overall, 90% of the mutations were located on exon 3, which contains the olfactomedin homology [7, 8].

According to Souzeau’s study, MYOC gene mutations are associated with disease severity and are more commonly found in advanced POAG [9]. Elahi et al. studied mutations of the MYOC gene in Primary Congenital Glaucoma (PCG) in the Iranian population and found it as an insignificant cause [10]. Therefore, the current study was designed to prove or rule out the disease causative role of MYOC gene mutations, using the Polymerase Chain Reaction-Single Strand Conformation Polymorphism (PCR-SSCP) method, among POAG patients of Khorasan district. This study also compared clinical features between the mutant and the wild group in the study population. Lack of information about genetic susceptibility of POAG in the Iranian population and ethnical differences were reasons for performing the current study.

## METHODS

In this case-control study, 70 patients with POAG, who were referred to Khatam-Al Anbia eye hospital and 70 healthy blood donors were enrolled from the Khorasan province of Iran in a study (project code:86626-MUMS) from January 2009 to September 2012. Simple non-probability sampling method was used to select the healthy control group and patients, who were referred to Khatam-Al Anbia eye hospital. This hospital is a tertiary center of Khorasan province, Iran, and participants also referred from other provinces.

The diagnosis of POAG was based on elevated Intraocular Pressure (IOP), abnormal perimetry changes, and an increased Cup to Disc Ratio (CDR) in the presence of a normal gonioscopy, which were diagnosed by an ophthalmology specialist. The exclusion criteria of the study were as follows: history of ocular trauma, uveitis, abnormal gonioscopy, and history of previous eye surgery or laser iridectomy. Slit lamp and fundus examination were done for all patients and visual acuity, Cup to Disc Ratio (CDR), and IOP were recorded by Goldman applanation tonometry (Haag Striet, Swiss) [2]. The controls (without a history of glaucoma) were recruited from the General Ophthalmology Clinic of the hospital, and were detected to have no ocular or systemic diseases, other than simple refractive errors. An informed consent was signed by all patients and controls. Age, gender, uncorrected Visual Acuity (VA), IOP and CDR of patients, and the healthy group were recorded for analysis.

Peripheral blood sample of each subject was obtained, and by using a commercially available kit (Biogene Company, Iran), according to the manufacturer’s instructions, genomic DNA was extracted. Genotyping of MYOC gene polymorphism was done by the PCR-SSCP method, as explained in previous studies [11, 12]. Three exons of the MYOC gene were amplified by the Polymerase Chain Reaction (PCR), using the single-strand primer for exons (Table 1).

The sizes of the single-stranded fragments were compared by electrophoresis on a neutral polyacrylamide gel. The proliferated bands were detected by silver staining, and the results were interpreted.

This study received ethical approval from the ethics committee of Mashhad University of Medical Sciences and written informed consent was signed by all study subjects. Patients’ name and results of the test were secured by a code.

The chi-square and Fisher’s exact test were used to compare genotypic frequencies of the study groups. Moreover, independent samples t-test and logistic regression test were performed. Statistical analysis was conducted using the SPSS software for Windows (version 15.0, SPSS, Chicago, IL). P-values of less than 0.05 difference were considered significant.

## RESULTS

Seventy patients, including 52 (75.1%) males and 18 (24.9%) females, with mean ± Standard Deviation (SD) age of 56.7 ± 18.6 years and 70 controls, including 35 (50%) male and 35 (50%) females, with mean ± SD age of 27.2 ± 6.9 years were enrolled in the study. The results of initial and final IOP, CDR, and VA studies revealed that IOP decreased significantly due to medical and surgical intervention in the patient group (P-value < 0.001). In spite of significantly decreased IOP, the CDR was increased significantly (P-value < 0.001). Comparison of initial and final VA of the patients showed that right eye vision was not different (P-value = 0.036) while the left eye vision was significantly decreased between initial and final VA measurement (P-value = 0.005).

**Table 1:** **Myocilin Gene Primers, pair’s Sequences and Length of Amplified Sequences**

Primer Name	Primer Sequence	Amplified Sequence Length
Myo-1Fa	5'-CCTCACGTGGCCACCTCTGTC-3'	554 bp
Myo-1Ra	5'-GGTTTCCAGCTGGTCCCGCTC-3'	554 bp
Myo-1Fb	5'-ATAACTTACAGAGAGACAGCAGC-3'	494 bp
Myo-1Rb	5'-CTCTAGGAGAAAGGGCAGGCAG-3'	494 bp
Myo-2F	5'-GCCGGCAGCCTATTTAAATGTC-3'	404 bp
Myo-2R	5'-CCTGCTCTGACAAGGGAACAG-3'	404 bp
Myo-3Fa	5'-GCTGTCACATCTACTGGCTCTG–3'	736 bp
Myo-3Ra	5'-GTCATAAGCAAAGTTGACGGTAGC-3'	736 bp
Myo-3Fb	5'-TGGCACCTTGTACACCGTCAGC-3'	766 bp
Myo-3Rb	5'-CTCTTAAGCAAAGATTCCCACAAAG–3'	766 bp

**Myo: Myocilin, F: Forward primer, R: Reverse primer, bp: base pair.**

Genetic Analysis

Two loci in exon 1, one locus in exon 2, and two loci in exon 3 were evaluated for MYOC gene mutation. The result of genotyping showed that there was a statistically significant difference in the MYOC gene mutation between the study groups (40% versus 11.5%, respectively, P-value < 0.001, OR = 5.1, CI95% for OR: 2.1 to 12.4). Moreover, the frequency of mutations in exon 1a and 3a were significantly higher in the patient group compared to the control group (15.7% versus 0% P-value = 0.001, OR: 27.2, CI95% for OR: 1.5 to 472.2 and 11.5% versus 2.8%, P-value = 0.049, OR: 4.3, CI95% for OR: 0.9 to 21.4, respectively) yet the difference between the frequency of 1b and 3b exon mutations was insignificant. Also, there was no frequency difference between the study group in exon 2 (Table 2).

**Table 2:** **Genetic Variation Between Patients and the Control Group**

Locus of mutation	Mutation	Patients	Controls	P value*	*P value (Adjusted by age)	OR*	CI 95 for OR
1a	Positive	11 (15.7)	0 (0)	0.001	0.006	27.2	1.5 to 472.2
	Negative	59(84.3)	70 (100)	-	-	-	
1b	Positive	6 (8.5)	2 (2.8)	0.14	0.07	3.1	0.6 to 16.3
	Negative	64 (91.5)	68(97.2)	-	-	-	-
2	Positive	0 (0)	0 (0)	-	-	-	-
	Negative	70 (100)	70 (100)	-	-	-	-
3a	Positive	8 (11.4)	2 (2.8)	0.049	0.12	4.3	0.9 to 21.4
	Negative	62(88.6)	68(97.2)				
3b	Positive	9 (12.9)	4 (5.7)	0.14	0.28	2.4	0.7 to 8.3
	Negative	61(87.1)	66(94.3)				

Data are presented as No (%). n: number; %: percentage; OR: odds ratio; CI: Confidence interval.

* Statistically analyzed using Chi-Square, univariate general linear models and logistic regression tests.

Genetic Variation and Clinical Course

The visual acuity, IOP, and CDR were compared between wild-type and mutant type of the studied gene in the patient group. The mean ± SD of VA (Log Mar) in all cases was 0.43 ± 0.45 for the right eye and 0.57 ± 0.61 for the left eye of patients, who carried the wild-type gene. Furthermore, mean ± SD of VA (Log Mar) was 0.48 ± 0.48 and 0.36 ± 0.43 for the right and left eyes, respectively, in patients with the mutant type of the selected gene. These differences were not statistically significant between groups (P-value = 0.7 for the right eye and P-value = 0.17 for the left eye) (Table 3).

The mean ± SD of the recorded IOP under topical anti-glaucoma drops in all cases was 15.9 ± 13.5 (mmHg) and 22.7 ± 10.1 (mmHg) for the right and left eyes in the patients carrying wild-type genotype and also 22.5 ± 1.5 (mmHg) and 19.9 ± 9.1 (mmHg) for the right and left eye of the patients with mutant type genotype, respectively. These differences were not significant between the groups (P-value = 0.34 for the right eye and P-value = 0.32 for the left eye). Table 4 represents the comparison of IOP between mutant and wild-type genotypes in each exon, separately.

Overall, mean ± SD of CDR was 0.7 ± 0.27 and 0.66 ± 0.26 for the right and left eyes of patients carrying wild-type genotype, respectively. In addition, mean ± SD of CDR was 0.75 ± 0.23 and 0.65 ± 0.24 for the right and left eyes in patients with the mutant type. The cup to disc ratio differences between studied groups were not statistically significant (P-value = 0.34 for the right eye and P-value = 0.32 for the left eye) (Table 5).

**Table 3:** **Comparing the Visual Acuity (Log MAR) between Participants Carrying the Mutant and Wild-type Sequence**

Site of changes	Mutant Mean ± SD	Wild type Mean ± SD	P value *
1a			
**Right eye**	0.66 ± 0.65	0.41± 0.41	0.15
**Left eye**	0.37 ± 0.60	0.51 ± 0.55	0.51
1b			
**Right eye**	0.64 ± 0.69	0.43 ± 0.43	0.33
**Left eye**	0.72 ± 0.66	0.47 ± 0.51	0.35
3a			
**Right eye**	0.78 ± 0.61	0.41 ± 0.43	0.09
**Left eye**	0.53 ± 0.65	0.49 ± 0.55	0.86
3b			
**Right eye**	0.28 ± 0.25	0.47 ± 0.48	0.30
**Left eye**	0.40 ± 0.34	051 ± 0.58	0.59

VA: visual acuity; Log MAR: Logarithm of the Minimum Angle of Resolution; SD: Standard Deviation.

* Statistically analyzed using Independent T test.

**Table 4:** **Comparing the** **Intraocular Pressure** **between Participants Carrying Mutant and Wild-type Sequence**

Site of changes	Mutant IOP (Mean ± SD)	Wild type IOP (Mean ± SD)	P value *
1a			
**Right eye**	18.38 ± 6.78	25.69 ± 13.68	0.14
**Left eye**	18.38 ± 5.26	22.18 ± 10.86	0.33
1b			
**Right eye**	27.0 ± 8.88	24.48 ± 13.53	0.68
**Left eye**	27.80 ± 13.04	21.08 ± 9.98	0.16
3a			
**Right eye**	24.83 ± 17.56	24.68 ± 12.79	0.97
**Left eye**	22.33 ± 6.71	21.58 ± 10.70	0.86
3b			
**Right eye**	20.13 ± 9.94	25.41 ± 13.53	0.29
**Left eye**	18.50 ± 10.67	22.16 ± 10.28	0.35

** IOP:** **intraocular pressure; mmHg: millimeter of mercury; SD: Standard Deviation.**

* **Statistically analyzed using Independent T test. **

**Table 5:** **Comparing the Cup to Disc Ratio between Participants Carrying the Mutant and Wild type Sequence**

Site of changes	Mutant CDR Mean ± SD	Wild type CDR Mean ± SD	P value *
1a			
**Right eye**	0.77 ± 0.23	0.70 ±0.26	0.48
**Left eye**	0.71 ± 0.21	0.64 ± 0.26	0.51
1b			
**Right eye**	0.27 ± 0.30	0.71 ± 0.25	0.96
**Left eye**	0.64 ± 0.32	0.65 ± 0.25	0.87
3a			
**Right eye**	0.75 ± 0.27	0.71 ± 0.25	0.73
**Left eye**	0.65 ± 0.28	0.65 ± 0.25	0.94
3b			
**Right eye**	0.75 ± 0.18	0.70 ± 0.26	0.68
**Left eye**	0.63 ± 0.22	0.66 ± 0.26	0.81

**Data in table are presented as Mean ± SD.** **CDR: cup to disc ratio; SD: Standard Deviation.**

* **Statistically analyzed using Independent T test.**

## DISCUSSION

The current study was designed to prove or rule out the disease causative role of the MYOC gene mutations by screening the MYOC gene, using SSCP analysis and comparing clinical features among POAG patients in the Khorasan district. The SSCP analysis was used to screen healthy and POAG subjects in every three exons of the GLC1A gene (MYOC gene). The results revealed that the frequency of MYOC gene polymorphisms in patients was higher than the control group. A possible explanation for the aggressive nature and weak response to treatment of these patients is the inheritance of the mentioned polymorphisms. According to the obtained results, the mean CDR was 0.7 in spite of full medication in nearly all patients. On the other hand, although all of the patients were Iranian, they were from different ethnicities and districts, as Khatam-Al Anbia eye hospital is a referral center in the east of Iran.

Genetic factors are responsible for the progression and response to treatment in many diseases. In glaucoma, genetic factors are important in disease progression or stability, yet due to variation in the results of studies on different ethnic groups and societies, there is no agreement on the role of genetic factors in POAG susceptibility [3-6].

Several mutations have been reported in different ethnic groups for POAG. In 1999, Finger et al. reported that the mean frequency of POAG mutations in different ethnic groups was 3% [8]. Based on the current study, the frequency of MYOC gene polymorphism was higher than other populations and these differences were statistically significant at the 1a and 3a position. So far, two other studies on genetic susceptibility of glaucoma have been done on the Iranian population. One of these studies screened the common CYP1B1 mutations in Iranian POAG patients, which found CYP1B1, as an effective cause in POAG subjects, mainly in juvenile glaucoma. Seven patients with POAG (11.1%) were carriers of CYP1B1 mutations whereby the majority, including five, were in the juvenile-onset group (5/21) and the rest were in the late-onset group (2/42) [13]. Another study by Elahi et al. was conducted to test the frequency of MYOC gene mutations in Iranian patients with PCG, in which none of the patients had mutations in this gene. The authors concluded that in Iranian PCG patients, the mutation of this gene could not be the main cause [10].

The influence of the disease course and response to treatment by the presence or the type of mutation has been reported in several studies. n the study of Graul et al., the rates of laser trabeculoplasty and surgery in POAG patients with a GLN368STOP MYOC mutation were similar with POAG patients without this mutation [14]. In contrast, in another study by Craig et al., the rate of filtration surgery in glaucoma patients with GLN368STOP mutations was higher than those without the mutations [15].

Souzeau et al. compared advanced glaucoma with less advanced cases and found a higher frequency of MYOC mutations in an Australasian disease registry. The frequency of MYOC mutations in glaucomatous patients with severe visual field loss was significantly higher than early glaucomatous patients [9]. In contrary, based on the results of the current study, there was no association between clinical signs of glaucoma and the presence of MYOC gene polymorphisms, according to the PCR-SSCP method. The IOP, CDR, and VA were not statistically different between the participants carrying wild-type and mutant type of the MYOC gene. Therefore, the findings may question the theory of the effect of genetic factors on the course of POAG. However, to reach more conclusive results more studies with larger sample sizes, stronger design, and racial diversity are needed. Furthermore, for more precise estimation of the association between gene mutations and susceptibility of diseases, the obtained results by PCR-SSCP should be confirmed by gene sequencing. Moreover, small sample size and not-completed complementary demographic data of case and control participants were other limitations of the present study. Also, the difference in patient and control group’s age was another limitation of the present study. 

## CONCLUSIONS

The results of the current study showed that there was a statistically significant difference in MYOC gene mutation between patient and healthy groups, especially in exon 1a and 3a. Also, there was no association between clinical signs of the disease and the presence of MYOC gene polymorphisms. The findings should be confirmed by more specific methods, such as gene sequencing. Because of the role of genetic variations in increasing susceptibility to glaucoma, it is necessary to find other variations and mutations as well.
